# Annotations

**Published:** 1907-08-31

**Authors:** 


### ANNOTATIONS.

### The Prevention of Cardiac Valvular Disease.

Dr. Caton, of Liverpool, has frequently urged his views on this question on the attention of the profession, and though the number of his professed disciples does not seem to increase, he continues his crusade with praiseworthy perseverance. It is almost impossible to exaggerate the amount of suffering and distress which are produced by cardiac valvular disease, and anything which affords a chance of reducing even a fractional part of this ought to be eagerly and steadily adopted. Dr. Caton's programme includes the diminution of pain and fever in acute rheumatism by the use of the salicyl compounds; absolute quiet and rest of mind and body, with sedatives, if necessary, to secure these ends; fairly full doses of potassium or sodium iodide to aid absorption of inflammatory products and to reduce blood pressure; and the use of small blisters over the upper four left intercostal spaces, by which means, he argues, it is possible to stimulate the trophic and vasomotor nerves of the heart without affecting the cardiac muscle. In addition he insists upon the necessity for rest, continued for six or eight weeks, or even for a still longer period. Another contribution to this subject has been made by Dr. C. O. Hawthorne, who points out that a large proportion of cardiac lesions occur in the early years of life, when rheumatism is common in its non-articular forms. These forms of the disease are not known to the public as "rheumatism," and hence the risk of heart disease and the necessity for rest during such apparently minor ailments are not recognised. Dr. Hawthorne, therefore, argues that it is the duty of any practitioner who discovers evidences of rheumatism in any family to warn the parents of the risks which are attached even to the slighter illnesses of their children. In this way, he contends, early rest might be secured, and thus a certain number of children might be saved from the disaster of organic heart disease.

### The Debate on the Early Notification of Births Bill.

In our issue of July 6 we stated generally what we regarded as the attitude which the profession should adopt towards the provisions of this Bill. Briefly, we contended that the medical attendant was the private adviser of the patient, and that it was an invasion of that relationship, and, therefore, injurious to the public interest, to compel him to disclose information which he had gained in the discharge of his professional obligations. It is, we think, much to be regretted that the medical authorities who had an opportunity to present the case for the profession to the Government and to Parliament, did not insist on this position, but laid emphasis rather on the omission of any proposal to pay a notification fee. Even as it was the whole matter was bungled during the discussion in committee. The opposition of certain medical members of the House seems to have been bought off by an amendment exempting medical practitioners from the compulsory clauses of the Bill. Upon the discussion of this amendment, however, so badly was the medical view of the matter presented that the sense of the House was obviously against the proposal. Speaker after speaker referred contemptuously to the demand of the doctors for a fee, and when one of the medical members urged that it was not the absence of a "fee," but the existence of a "penalty" for failing to notify, to which the profession objected, his remarks, not unnaturally, were received with scornful laughter. Only, so far as we have seen, in a single instance, was reference made to the principle that the private medical adviser of the patient ought not, in the general interest, to be compelled publicly to disclose professional confidences. In the end, the amendment, the acceptance of which had been made the price of the withdrawal of opposition to the advance of the Bill, was lost. So that those who claimed to voice the view of the profession to the House appeared both as Parliamentary bunglers and as the representatives of purely sordid interests. Medicine may well pray to be delivered from some of her friends.

### Strychnine in "Tabloids."

A very important case was decided a few days ago by Mr. Mead at the West London Police Court. The question at issue was, whether an offence against the Pharmacy Act had been committed by the sale of "tabloids" of Easton's syrup without any entry of the sale, and without the signature of the purchaser, in the appropriate "Poisons Book" prescribed by the Act. The "tabloids," it was admitted, contained strychnine, and strychnine, according to law, must, when sold, not only be labelled "poison," but the sale must be registered as just described. It was also admitted that, in the instance in question, no such registration was made. In defence it was pleaded that such omission, while possibly constituting a technical offence, was in harmony with the general custom adopted by chemists and druggists throughout the country. Hence a merely nominal penalty was suggested as sufficient to meet the position. The magistrate, however, refused to accept this view. He held that a substantial contravention of the Act had taken place, and inflicted the full penalty of £5. If the custom of the trade was as stated, it was all the more important to emphasise the gravity of the offence. The objects of the special provision demanding the purchaser's signature were, first to impress on the purchaser the fact that the translation carried serious risks, and, secondly, to enable the purchaser to be traced should the poison be used for any illegitimate purpose. Behind the legal aspects of the case the question may be raised whether it is advisable to offer freely to the public powerful, and even poisonous, medicinal preparations in such an accessible and seductive form as compressed tablets. There is surely a lesson in the fact that the event which led to the above trial was the death of an infant after swallowing a number of "tabloids" set free by the accidental breaking of the bottle.

